# Enhancement of Gingival Tissue Adherence of Zirconia Implant Posts: In Vitro Study

**DOI:** 10.3390/ma14020455

**Published:** 2021-01-19

**Authors:** Alexandra Zühlke, Michael Gasik, Khalil Shahramian, Timo Närhi, Yevgen Bilotsky, Ilkka Kangasniemi

**Affiliations:** 1 School of Chemical Engineering, Aalto University Foundation, 00076 AALTO Espoo, Finland; 2 Department of Prosthetic Dentistry and Stomatognathic Physiology, Institute of Dentistry, University of Turku, 20500 Turku, Finland; khalsha@utu.fi (K.S.); timnar@utu.fi (T.N.); 3 Turku Clinical Biomaterials Center (TCBC), University of Turku, 20500 Turku, Finland; 4 Welfare Division, Oral Health Care, 20500 Turku, Finland; 5 Seqvera Ltd. Oy, 00290 Helsinki, Finland; seqvera.research@gmail.com; 6 ID Creations Oy, 20760 Turku, Finland; ilkka.kangasniemi@idcreations.fi

**Keywords:** zirconia, dental, abutment, gingiva, dynamic mechanical analysis, in vitro, in silico, modeling

## Abstract

Prevention of bacterial inflammation around dental implants (peri-implantitis) is one of the keys to success of the implantation and can be achieved by securing the gingival tissue-abutment interface preventing penetration of bacteria. Modern dental practice has adopted zirconia abutments in place of titanium, but the adhesion of gingival tissue to zirconia is inferior to titanium. The aim of this study was to assess and improve the adhesion of mucosal tissues to zirconia posts using sol-gel derived TiO_2_ coating following dynamic mechanical testing. The posts were cultivated with porcine bone-gingival tissue specimens in vitro for 7 and 14 days and then subjected to dynamic mechanical analysis simulating physiological loading at 1 Hz up to 50 μm amplitude. In parallel in silico analysis of stresses and strains have been made simulating “the worst case” when the fixture fails in osseointegration while the abutment still holds. Results show treatment of zirconia can lead to double interface stiffness (static shear stiffness values from 5–10 to 17–23 kPa and dynamic from 20–50 to 60–125 kPa), invariant viscostiffness (from 5–35 to 45–90 kPa·s^α^) and material memory values (increased from 0.06–0.10 to 0.17–0.25), which is beneficial in preventing bacterial contamination in dental implants. This suggests TiO_2_-coated zirconia abutments may have a significant clinical benefit for prevention of the bacterial contamination.

## 1. Introduction

A dental implant system can be used to restore a missing tooth or teeth. It is made of three parts: the implant, an abutment and a crown [1]. The implant is placed on the jawbone where it is supposed to osseointegrate with the bone tissue and the crown is attached on the top using a screw or cement. The abutment as the mid-part is placed on the implant with different locking configurations for the purpose of connecting the implant through soft gingival tissues to the oral environment. The abutment must have firm and stable adherence with gingival tissues to prevent a bacterial passage down to the implant. A tight “good” seal between the soft tissue and the abutment is required for long term success of a dental implantation [2,3]. This is to prevent bacterial inflammation in the adjacent tissues, which may lead to bone resorption and implant failure. The force or torque, which corresponds to this “good seal” between the gingiva and abutment, is difficult to quantify—most of the numerical data are reported on torque values used for characterization of the abutment-screw fixation “quality” [4]. The abutment-tissue attachment is exposed to different mechanical stresses from swallowing mastication and parafunctions (grinding, clenching). The mastication frequency depends on the individual (sex, age, habits, food type) but is determined to be around 1 Hz. This is different from frequencies seen in animal models, for example, porcine about 2–3 Hz [5], so animal data might not be directly translatable.

The risk of inflammation is increased if bacteria can form a biofilm on the abutment surface [6,7,8]. Soon after implantation there is a competition between the bacteria and the epithelial cells to attach to the abutments surface [9]. Both the epithelial cells and the bacteria use the same molecules to bind to the surface and they can both inhibit each other’s attachment. If the bacteria can form a biofilm on the abutments surface it matures quite easily, since it is hard to clean [9,10]. The accumulation of bacteria may lead to inflammation, which can also then spread to peri-implant gingival tissues and the bone, which then leads to bone resorption and might eventually lead to loosening of the implant [9,11].

The common goal is to reduce the risk of infection, which can be theoretically achieved by controlling the local environment to be less favorable to bacteria (such as adjustment of surface topography and hydrophilicity [12,13]), by using antibacterial medicine or by preventing the bacterial contamination before biofilm could form on the abutment or implant surface [8,14]. Ceramic abutments are gaining now popularity due to favorable esthetical reasons. Stabilized zirconia (based on zirconium dioxide, ZrO_2_) is a very good alternative ceramic material to be used in abutments in place of titanium, as it is chemically stable, biocompatible and has a high wear resistance [15,16]. It is also suitable from the patient’s perspective being odorless and tasteless with adjustable color [17,18,19]. The downside of zirconia compared to titanium is that studies have shown that epithelial cell attachment is inferior on zirconia surface compared to titanium [11,13,20]. The aim of this study was to examine the effect of TiO_2_ coatings on zirconia posts to enhance the soft tissue adherence, evaluated by dynamic mechanical analysis (DMA) combined with computer in silico simulation.

## 2. Materials and Methods

### 2.1. Zirconia Materials

Yttria-stabilized zirconia (3Y-TZP; CeraPost^®^, Komet Dental, Gebr. Brasseler GmbH and Co., Lemgo, Germany) endodontic posts (*n* = 12) of 10 ± 1 mm length and 1.95 ± 0.05 mm diameter were used in this study to function as implants that were inserted in porcine gingival tissue, as described by Shahramian et al. [13], to simulate the intraoral soft tissues attachment to zirconia abutments. They do not mimic the full implant construction, but in this study the target was mainly on the abutment–gingiva interface.

The posts were prepared as described in [13] by cutting and cleaning in ultrasonic baths (VWR International Oy, Helsinki, Finland) of acetone and ethanol subsequently for 5 min each. Two experimental groups were made, uncoated zirconia (control) and zirconia posts coated with sol-gel derived TiO_2_. The sol–gel solution was made by dissolving tetraisopropyl orthotitanate [Ti(OCH(CH_3_)_2_)_4_] in 95% ethanol and mixing it with a solution of ethanol, HNO_3_ and ultrapure water. The resultant solution was left to age at room temperature for 24 h. The TiO_2_ coatings were prepared by dipping the specimens into the solution and then withdrawing them at a speed of 0.3 mm/s following the heat treatment at 500 °C for 1 h and were cleaned ultrasonically again [13]. The posts were sterilized by autoclaving for 20 min at 121 °C.

**Figure 1:**
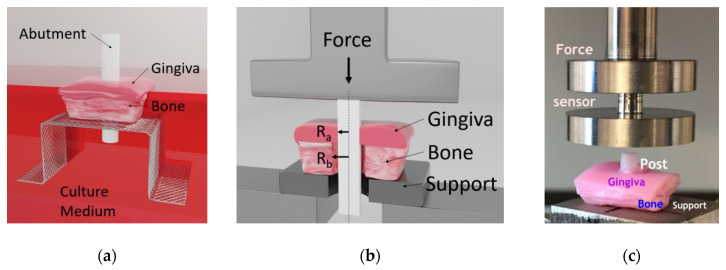
Experimental setup: (**a**) arrangement of the abutment post and tissues for cultivation; (**b**) mechanical setup of the specimen in the dynamic mechanical analysis (DMA) sample holder; (**c**) photo of the actual specimen in the sample holder.

On the contrary to the previous study [13], these posts were implanted not in stand-alone gingival tissue but in the full-thickness porcine tissue comprising both gingiva and the underlying bone in a way that the integrity of the interface between the bone and gingiva was maintained (Figure 1a,b). The samples were obtained by dissection of the mandible of freshly slaughtered pigs with a surgical saw as described by Shahramian et al. [13] as ~8 × 8 mm blocks (bone and gingiva were cut together) and promptly rinsed in PBS supplemented with penicillin, streptomycin, and amphotericin B. The average thickness of the gingiva was 3.30 ± 0.46 mm and the underlying bone 1.48 ± 0.34 mm as measured by a non-contact laser micrometer (Metralight Inc., Burlingame, CA, USA) with ±1 μm precision before further tests. All the tissues were checked to be viable before further manipulations. Before the placement of the posts, the hole in the bone was made with a precision drill from the bone side (in order not to penetrate the gingiva), resulting in the hole diameter of 3.20 ± 0.22 mm. The gingival parts were then pierced using an 18G needle [13] to mimic the surgical wound created clinically during abutment placement. Thus, the hole in the bone was made slightly larger (by ~1.5 mm) than the hole in the gingiva making the bone a natural support for the shear bending. This ensures that the soft tissue remains attached to the bone in the lateral direction and the zirconia post simulates abutment in the “worst case” scenario, when the implant (fixture) has completely lost contact with the bone and the soft tissue penetrating abutment part can move freely. Therefore, good adherence of the post with the tissue would result in higher stresses needed to deform and displace the tissue to the same extent compared to control.

The placed posts were individually cultured at an air/liquid interface in 6-well plates containing culture medium (Eagle’s minimum essential medium EMEM M-2279) supplemented with 10% fetal bovine serum, 100 U/lg penicillin, streptomycin 100 lg/mL, and 200 mM L-glutamine. The specimens were incubated at 37 °C in a 5% CO_2_ environment with the culture medium changed every 24 h up to 7 and 14 days (three samples with a non-coated implant and three with a coated implant) in culture (Figure 1a).

### 2.2. Dynamic Mechanical Analysis

Analysis of the adherence of the posts to the gingiva was carried out using the model-free invariant Biomaterials Enhanced Simulation Testing (BEST) [21] method realized in dynamic mechanical analysis setup (Seqvera Ltd., Helsinki, Finland). The tests were done with a dynamic mechanical analyzer DMA242E “Arthemis” (Netzsch Gerätebau GmbH, Selb/Bayern, Germany) using a customized sample holder at 37 °C and 1 Hz frequency (Figure 1c) in a strain-controlled compression mode from 5 to 50 μm (resolution ±0.25 nm), which covers the conventional physiological range of displacements and frequencies of the dental abutments and implants as reported in [22,23]. All the samples’ dimensions were measured with a non-contact laser micrometer (Metralight Inc., Burlingame, CA, USA) with ±1 μm precision.

The samples underwent automatic preconditioning (guided by the embedded DMA software (Proteus 6.1, Netzsch Gerätebau GmbH, Selb/Bayern, Germany)) for ~5 min before the test started for the signal stabilization to eliminate inertial disturbances and possible effects of initial specimen misalignment. The force and displacements were measured via the same single probe, subtracting the empty system and the sample holder calibrations made before the tests. All tests were non-destructive ones so the posts remain in contact with the tissue afterward whereas they might undergo some irreversible deformation (creep). In this work the data of forces and displacements in the setup shown in Figure 1c were obtained directly from DMA after the test without pre-processing or modification. Forces and displacements were converted into stresses and strains, as shown below in Table 1. In the experiments, shrinkage or swelling of the soft tissues were not observed.

### 2.3. Experimental Data Processing

The tests described in the earlier study [13] were relying on using a thin plate mechanical theory with empirical coefficients not feasible for specimen type shown in Figure 1. In this work, the specimen geometry has required a different way of calculating stresses and strains as it cannot be approximated by common thin plate or beam theories. In the BEST approach [21,24] no material models needed to be assumed, so the strains and stresses were calculated directly (Table 1) as coming from the geometry of the setup (Figure 1b).

The strain definitions in Table 1 follow the common approach that the engineering strain is the change of the tangent of the angle (non-dimensional) due to applied shear force, whereas the true (logarithmic) strain is the change of this angle (in radians). There is sometimes a confusion in the literature about the definition of the shear strains, and in the authors’ opinion the definition used in experiments should always be reported in sufficient detail. For dynamic shear amplitude here, an additional factor of ½ is due to the average amplitude (max–min angle) around the mean static deformation at that time point [21,25]. The stiffness (static and dynamic values in kPa) in this case was calculated as the ratio of respective stress σ_i_ to true or engineering strain ε_i_ [26].

The BEST method of the post-processing results foresees an application of the time convolution integral to harmonic stress input [21,24,27,28,29], which in general results in the non-linear equation for dynamic strain as function of time *t*, frequency ω and stress σ_dyn_:

(1) $$\varepsilon_{\mathrm{dyn}}(t,{\omega ,\sigma }_{\mathrm{dyn}})=\frac{1}{\Gamma (\alpha )\times C_{\omega }}\overset{t}{\underset{0}{\int }}\;\frac{\sigma_{\mathrm{dyn}}\sin(\omega \tau )d\tau }{{(t-\tau )}^{1-\alpha }}$$

where *C*_ω_ is the dynamic viscostiffness (quasi-property in units of kPa·s^α^), α—dynamic material memory parameter (unitless). This equation is valid for any system and it does not require postulation of any model of the material, nor does it require linear elasticity or viscoelasticity assumptions to be valid [30]. BEST comprises time-convolution of the specimen loading history without a need to involve complex algebra (without using complex numbers) or artificial representations of a material (Maxwell, Burger, standard linear solid, Prony series, etc.) [21].

The *C*_ω_ and α values are time-invariant in a sense that they do not depend on the time of experiment. Equation (1) can be always numerically explicitly computed without the need for assumptions of linearity of *C*_ω_(ω,σ_dyn_) and α(ω). The values of memory parameter in Equation (1) must be non-negative to ensure causality principle, and in the range 0 < α < 1 they present the fading memory (zero means only short-time memory, approaching ideally elastic behavior, whereas unity means long-time memory linked to ideally viscous behavior) [27,28,29].

### 2.4. In Silico Modeling

Modeling of the experimental setup and the testing conditions was performed with COMSOL Multiphysics 5.3 software (COMSOL Inc., Burlington, MA, USA) in 3D space using an axially symmetric model of the tissues (gingiva and the bone). The materials properties were chosen as for a linear elastic material, but the values of stiffness were entered as a non-linear user function (1) from the obtained experimental DMA data. Zirconia posts were assumed fully rigid with respect to the tissues and they were not explicitly simulated, imposed rather as a boundary condition of the respective displacement transfer to the soft tissue. The basic conditions for simulation comprise a 50 μm displacement amplitude at 1 Hz frequency applied axially (along z-direction) to the post-tissue interface up to 300 s in the axial 2D symmetric model (this reflects the conditions of DMA tests during 5 min). No big differences between the engineering and true strains (Table 1) were found in the outcome values, so it was reasonably assumed that small strain limits are the correct assumption for this geometry setup, even the shear strains could be as high as 0.40–0.45.

### 2.5. Statistical Analysis

Statistical analysis of the data was performed first with direct outcomes data (stiffness values) and second with idempotent analysis [21,24] to extract invariant [26] values. For the first, unpaired median differences between controls (untreated) and coated zirconia post was analyzed and for viscostiffness Cohen’s d-value was calculated (the difference in means divided by the pooled standard deviation of the two samples) with 5000 bootstrap samples and bias-corrected and accelerated confidence intervals [31]. Converted data of biomechanical measurements representing viscous stiffness after time convolution were fitted with exponential function of memory values as it is theoretically predicted by idempotent analysis [21,24].

The presence of leverage points was detected with hat matrix diagonal components not falling under Stephen’s rule (these points were removed). Influence (outlier) points analysis was made by calculating Cook’s distances, and those data points exceeding the unity value (if any found) were removed. The consistency of regression coefficients was independently checked by application of the Theil–Sen estimator and the goodness of fit significance by modified Anderson–Darling test (data not shown). Heteroscedasticity of fitting residuals was estimated with the RUNS test and the residuals autocorrelation with the Durbin–Watson parameter. All the data below have passed these criteria, and obtained biomechanical invariant values were considered to be best linear unbiased estimators (BLUE).

## 3. Results

### 3.1. Stiffness and Invariant Values Analysis

**Figure 2:**
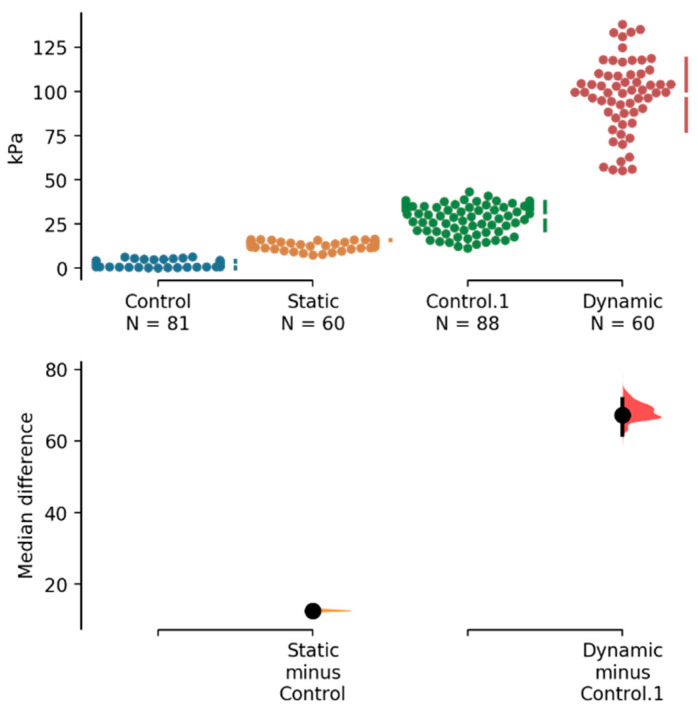
The median differences for two comparisons (static and dynamic stiffness) are shown in the above Cumming estimation plot. The raw data are plotted on the upper axes; each mean difference is plotted on the lower axes as a bootstrap sampling distribution. Mean differences are depicted as dots; 95% confidence intervals are indicated by the ends of the vertical error bars.

The stiffness (stress/strain ratio in kPa) values for static and dynamic loadings are compared in Figure 2. The unpaired median difference between control (=static stiffness at control) and static stiffness for coated posts is 12.6 kPa (confidence interval (95.0%) 11.4~13.1; *p* = 0.0). The p-values reported are the likelihoods of observing the effect sizes, if the null hypothesis of zero difference is true. This means that there is a statistically significant difference between the static loading reaction of coated and uncoated posts, the former being superior in terms of withstanding static loads to the same deformation degree. The unpaired median difference between Control.1 (=dynamic stiffness at control) and dynamic stiffness is much larger—67.4 kPa (confidence interval (95.0%) 61.6~71.8; *p* = 0.0 for the two-sided permutation *t*-test). Thus, dynamic stiffness for the treated (coated) post being much higher all the time indicate that the coated post “feels” more resistance to moving better than in uncoated control.

**Figure 3:**
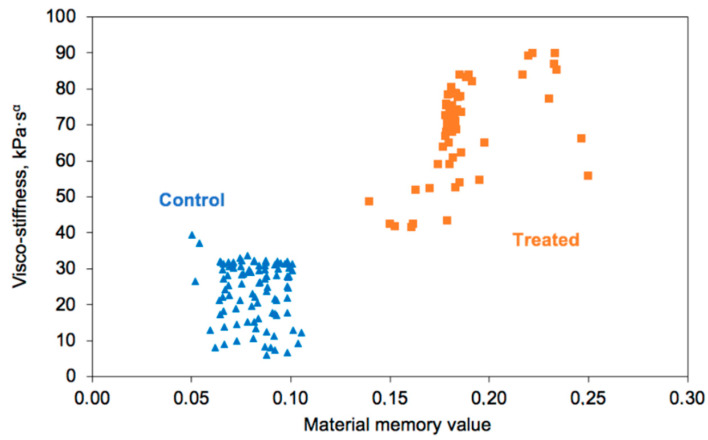
Data on dynamic viscostiffness and memory values for treated (coated) and control (uncoated) posts.

**Figure 4:**
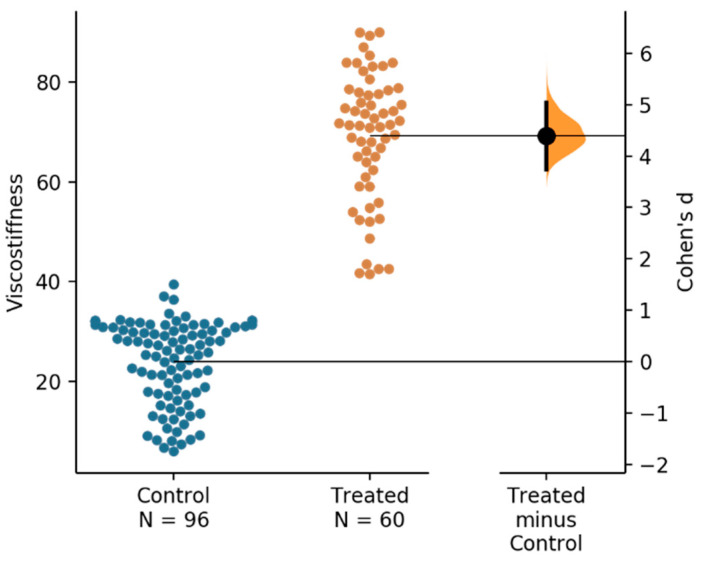
The Cohen’s d-value between control (uncoated) and treated (coated) samples viscostiffness (kPa·s^α^) shown in the Gardner–Altman estimation plot. Both groups are plotted on the left axes; the mean difference is plotted on floating axes on the right as a bootstrap sampling distribution. The mean difference is depicted as a dot; the 95% confidence interval is indicated by the ends of the vertical error bar.

Figure 3 shows the clusters of data of viscostiffness and memory value (α) for these two groups of the specimens. It is seen that these cases indeed present two distinct clusters and that the treated case shows more damping capacity (higher memory value). Statistical analysis of the viscostiffness is shown in Figure 4. It is clearly seen that with the unpaired Cohen’s d-value between control and treated groups is 4.39 (confidence interval (95.0%) 3.73~5.03; *p* = 0.0), indicating a significant difference.

The extraction of the data from the viscostiffness values show that the control specimens require about 2 s to fully react on dynamic stimulus (shorter memory < 0.10), whereas treated samples would need even about 12 min to completely recover from the one loading cycle (hence treated samples have a higher material memory ~0.20, meaning also its viscous component is significantly larger). These characteristic times can be associated with the Deborah numbers of the system as they reflect the “reaction to flow”: when the time of observation is significantly lower than the characteristic time, the system (post + tissue) appears to be less responsive to the stimulus.

### 3.2. Computer in Silico Simulation

**Figure 5:**
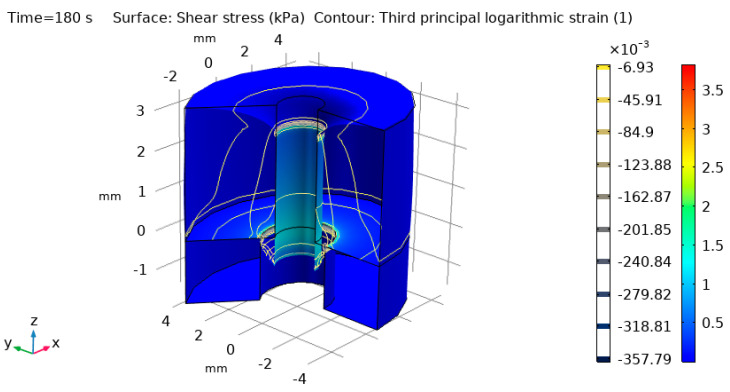
Snapshot of the simulation at 180 s from test start time with vertical loading along the Z–axis on the post surface (the post is not shown). Color scale shows shear stress τ_RZ_ and color contours third principal logarithmic (true) strain ε_3_. It is seen that most of the strain and stress accumulate at the top and the bottom of the post.

The snapshot of the simulation at 180 s after the start of the test is shown in Figure 5. This reflects the situation when the post was already loaded (180 full cycles at 1 Hz) in Z-direction and has undergone both static and dynamic deformations. If the tissue would have no creep effect present, the dynamic strains and stresses would have been the same for every loading cycle. As the tissue undergoes inelastic deformation (Δ*L*) with time, dynamic strains (Table 1) also change because the reference frame for strain calculation (Table 1) changes too.

**Figure 6:**
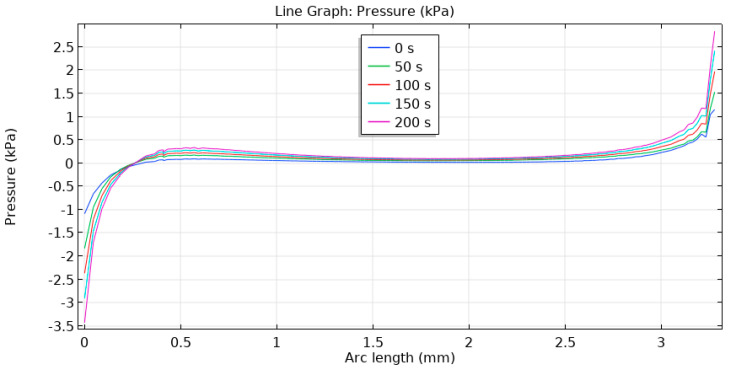
Pressure in the gingival tissue along the post interface. Color scale indicated respective times from the start of the experiment.

With the simulation it was also possible to calculate the hydrostatic pressure in the tissue, which is not possible to measure directly. This pressure in the tissue (assuming no intrinsic fluid movement) is mainly compressive at the top edge of the gingiva and is tensile at the bottom edge (Figure 6), but in all cases it does not exceed 4 kPa (<30 mmHg). Low pressure values are important to maintain if an implant exerts on surrounding soft tissues a pressure of 5–10 kPa (37–75 mmHg), as this still would allow hemocirculation even with some expected pain. Long-lasting pressures over >20 kPa (>150 mmHg) are to be avoided, as it is known to cause tissue necrosis and hypoxia following severe complications ultimately leading to the removal of the implant [32]. It is noteworthy that this pressure is not the same pressure usually estimated from masticatory loads due to different load transfer in that case.

**Figure 7:**
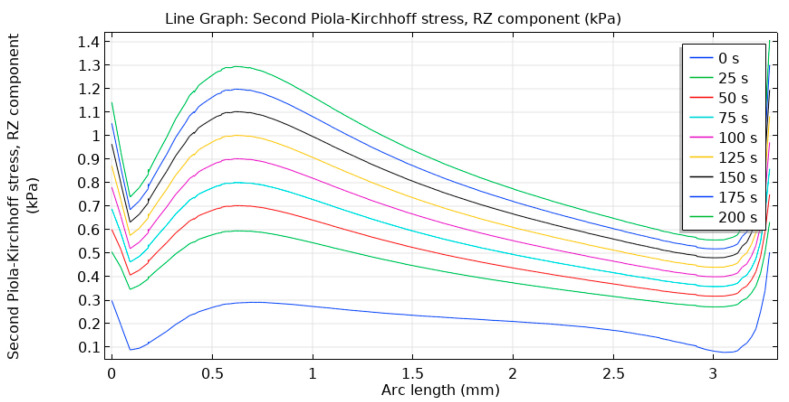
Shear stress τ_RZ_ in the gingival tissue along the post interface. Color scale indicated respective times from the start of the experiment.

Shear stress τ_RZ_ (shear RZ-component) has extrema at terminal points (top and the bottom of the post-tissue interface) and also at ~0.6 mm distance from the bottom of the contact of the post with the tissue (Figure 7). This is linked with the location of the maximal shear of the tissue vs. fixed point of the bone support. As seen, this shear stress tends to increase with time as the soft tissue undergoes some creep, compression and inelastic deformation.

## 4. Discussion

The goal of the zirconia–gingiva attachment is to prevent pathogens penetration and formation of the biofilm leading to peri-implantitis and implant loss. It is known that biofilms react in a different way to shear induced deformation [33], potentially leading to detachment, but the huge variety of different biofilms and their mechanical properties does not allow a single recommendation on the values one would need to remove the biofilm. Hence, it seems to be an easier way to prevent biofilm formation from the very beginning rather than attempting its removal at the later stages. In this sense, oral implant abutments having higher viscostiffness and memory values, and better attachment to the tissue are preferable.

In studies comparing the biological response of soft tissue to zirconia and titanium abutments, it was found that the zirconia abutment is associated with a significantly greater blood flow in the surrounding free gingival than for the titanium abutment [34]. It was therefore concluded that zirconia abutments promote better microcirculatory dynamics in peri-implant mucosa that is close to that of natural teeth [35]. Increase in blood circulation in peri-implant soft and hard tissue leads to improved immune response and that will further lead to decreased bone destruction [34], and in the light of this work assessment this could be also related to lower pressure imposed on the mucosa by the abutment.

The studies carried out in this work represent a combined in vitro–in silico model that attempts to simulate a zirconia abutment static and dynamic mechanical behavior using zirconia posts embedded into porcine gingival tissue. Porcine and human oral mucosal wounds are similar in terms of molecular composition and clinical and histological characteristics, which gives an indication that the behavior of the posts in porcine tissues could be similar to the expected behavior in humans [36]. Porcine mastication has, however, higher frequency (2–3 Hz) than human (~1 Hz) and due to anatomical differences, porcine in vivo data (if obtained) cannot be fully extrapolated to humans [5]. This calls for application of more relevant boundary conditions to approach more rational outcomes.

Experiments and simulations have shown that a significantly stronger gingival attachment was observed with TiO_2_ coated zirconia post compared to gingival attachment to uncoated posts under physiological dynamic loading relevant to human activity (~1 Hz up to 50 μm displacement). The differences obtained in this study between coated and uncoated zirconia posts were larger than observed previously in conditions [13] for similar zirconia posts cultivated in stand-alone gingival tissue only. This can be attributed to the use of a more natural structure of the samples in this study, since gingival tissue has retained its intimate contact with the underlying bone. These experimental boundary conditions are assumed to correspond better to clinical reality, as there was a “perfect” (natural) bone–gingival interface. In previous experiments [13] use of soft tissues alone has faced more scatter of the data as it was difficult to control sliding of the gingiva sample during mechanical testing. Furthermore, the degree of sliding and rotation of the sample in previous experiments was also dependent on the support (roughness, type, e.g., a steel plate or a mesh).

The most relevant findings of this study could be listed as follows:

- For in vitro mechanical testing of posts (and likely also full-size abutments) adherence to the gingival tissue it is useful to simulate the “worst case” when the implant fails in osseointegration but when abutment can still prevent pathogens penetration due to its sufficient tissue–abutment interface quality. It is reasonable to assume the abutments (posts) would equally perform in the normal case too when the tissue dynamic displacement is less than 50 μm in amplitude at 1 Hz.
- The tests are needed to be properly planned and executed in the right way to make the design of dental biomaterials with an enhanced clinical value. This concerns samples preparation, test conditions and results analysis.
- For the first time, invariant values (viscostiffness and material memory values) have been extracted from experimental data for these zirconia posts without application or assumption of a material model (viscoelastic or others). These values comprise time-convoluted data and are better predictors for materials performance comparison than traditional stiffness (stress/strain ratios), as the latter depends on the way stresses and strains are defined.
- Invariant values can be used in computer in silico simulations using a simple linear elastic material approach but with the values of elastic properties substituted with these values instead of some constants. This can simplify calculations and extend them into more realistic clinical cases with 3D implant placement planning and outcome estimation.
- An example using in silico simulation has demonstrated that coated zirconia posts would cause low hydrostatic pressure to gingival tissue, which is important to support blood circulation and regeneration of the surrounding tissues. Analogous non-coated zirconia posts would have failed earlier in the same conditions (even if they would have the same or lower pressure values).

This study also has some limitations. Besides the small number of samples used, there is an uncertainty in soft tissue properties, which vary depending on the location, storage, preparation, etc. This was addressed by cultivating control and treated post in identical conditions simultaneously, but this does not fully compensate for variations in absolute values of the gingival tissues mechanical properties. The values of stiffness and invariant memory values are likely to be different for another set of tissues even with the same abutment materials. However, as these values were compared to controls, it is reasonable to assume that the difference between control posts and coated posts has significant statistical differences, demonstrating coating effectiveness.

It would also be interesting to carry out a pull-out test or rotational test of the embedded posts, but it was not possible due to the limited sample size and geometry samples availability. Pull-out test data, however, are more rate-dependent, so it would require more samples to extrapolate the results to proper strains or deformation rates having clinical relevance [25]. Furthermore, soft tissues in the pull-out might undergo much more non-uniform deformation or even tearing so data quality would not be certain. A rotational test might be more representative, but it would analyze the loading effect in the different coordinate plane (XY plane in terms of Figure 5 definitions). Such rotation of abutment is much less likely to happen than a direct Z-axis compression common for mastication.

There are also diverse grades and compositions of stabilized zirconia for dental applications and it is not clear whether, for example, the same coating and coating technique would be equivalent for different zirconia materials with varied surface topology and composition [37,38]. For future studies, the stability of TiO_2_ coating vs. processing (sterilization, storage, handling) and deployment (placement) is very important to address. For long-term application, degradation of the coating might be a limitation, but it is likely to work with the healing abutment system when the fixture has been successfully osseointegrated.

## 5. Conclusions

The experimental in vitro and in silico study of coated (treated) and uncoated zirconia posts has shown that TiO_2_ coating via the sol–gel process has a statistically significant impact on improvement of the mechanical properties of the tissue–gingiva interface by improving the static and dynamic stiffness, the invariant viscostiffness, and material memory values in the “worst case” scenario. For the conditions used (1 Hz and up to 50 μm amplitude) coated posts have demonstrated:

- ~2-fold improvement in static stiffness (stress/strain ratio),
- ~3-fold improvement in dynamic stiffness (dynamic stress/strain ratio),
- ~2.5-fold increase in invariant viscostiffness and,
- ~3–4-fold increase in material memory value

Computer in silico analysis has shown that well-adhered coated posts even in this worst case do not generate local shear stresses in the gingival tissue over 5 kPa, which is a positive factor to be considered for the case of prevention of adjacent tissues necrosis or hypoxia due to insufficient blood supply. Altogether this suggests TiO_2_-coated zirconia abutments may have a significant clinical benefit for prevention of the bacterial contamination, which would also improve the success of the implant.

## Figures and Tables

**Table 1:** Stresses and strains at the abutment interface for the experimental setup ^1^.

Parameter	Static	Dynamic (1 Hz in This Work)
Shear stress	$\sigma_{\mathrm{stat}}=\frac{F_{\mathrm{st}}}{2\pi R_{a}H}$	$\sigma_{\mathrm{dyn}}=\frac{F_{\mathrm{dyn}}}{2\pi R_{a}H}$
True strain	$\varepsilon_{\mathrm{stat}}=(\frac{a}{\Delta R})$	$\varepsilon_{\mathrm{dyn}}=\frac{1}{2}\tan^{-1}\left[\frac{2a\times \Delta R}{{\left(\Delta R\right)}^{2}-{\left(\Delta L\right)}^{2}-\frac{a^{2}\times \left({\left(\Delta R\right)}^{2}+{\left(\Delta L\right)}^{2}\right)}{{\left(\Delta R\right)}^{2}-{\left(\Delta L\right)}^{2}}}\right]$
Engineering strain	$\varepsilon_{\mathrm{eng}.\mathrm{stat}}=(\frac{\Delta L}{\Delta R})$	$\varepsilon_{\mathrm{eng}.\mathrm{dyn}}=\frac{1}{2}\frac{a\times \Delta R}{{\left(\Delta R\right)}^{2}-{\left(\Delta L\right)}^{2}-\frac{a^{2}\times {\left(\Delta L\right)}^{2}}{{\left(\Delta R\right)}^{2}-{\left(\Delta L\right)}^{2}}}$

^1^*a* = experimental displacement amplitude (μm), Δ*L* = total observed static displacement (μm), Δ*R* = distance between the diameter of the hole in the supporting bone and diameter of the post (μm), *F*_st_ = applied static force on the post, N, *F*_dyn_ = applied dynamic force amplitude applied on the post, N, *H* = height of the implanted post in contact with soft tissue (μm), *R*_a_ = radius of the post (μm).

## Data Availability

Original data are available on request from the corresponding authors.
